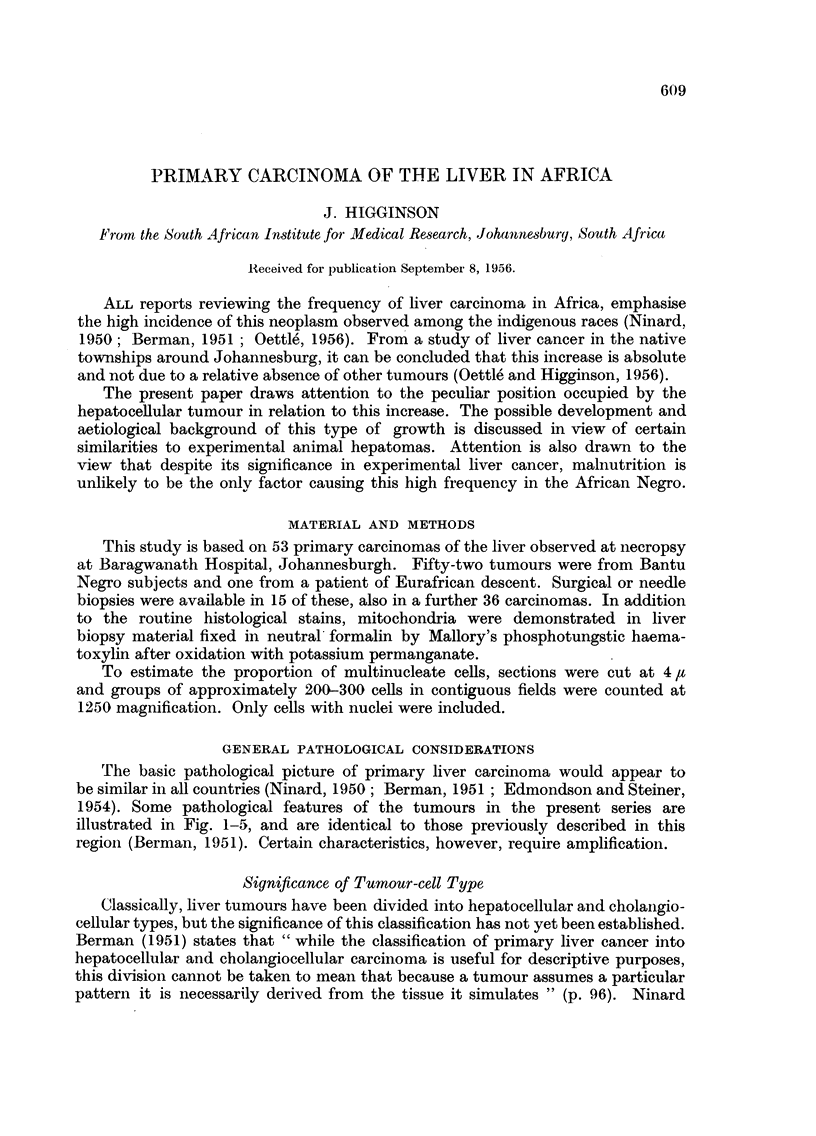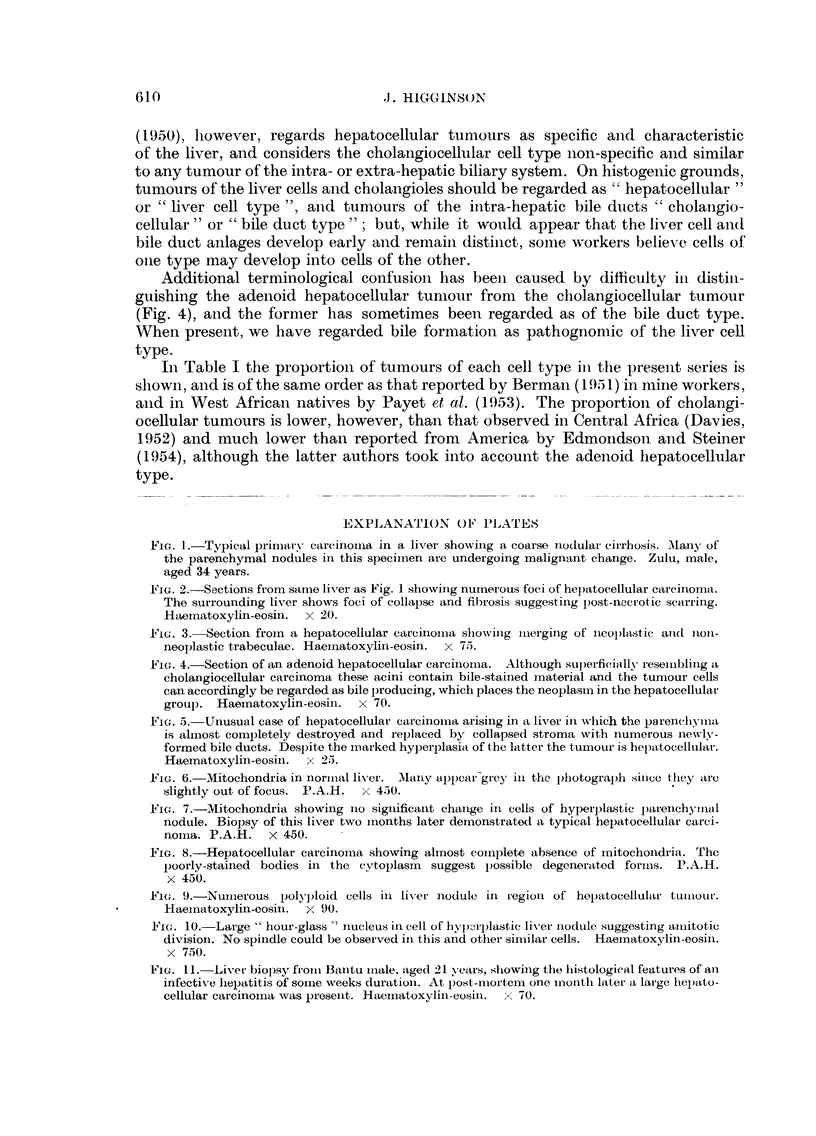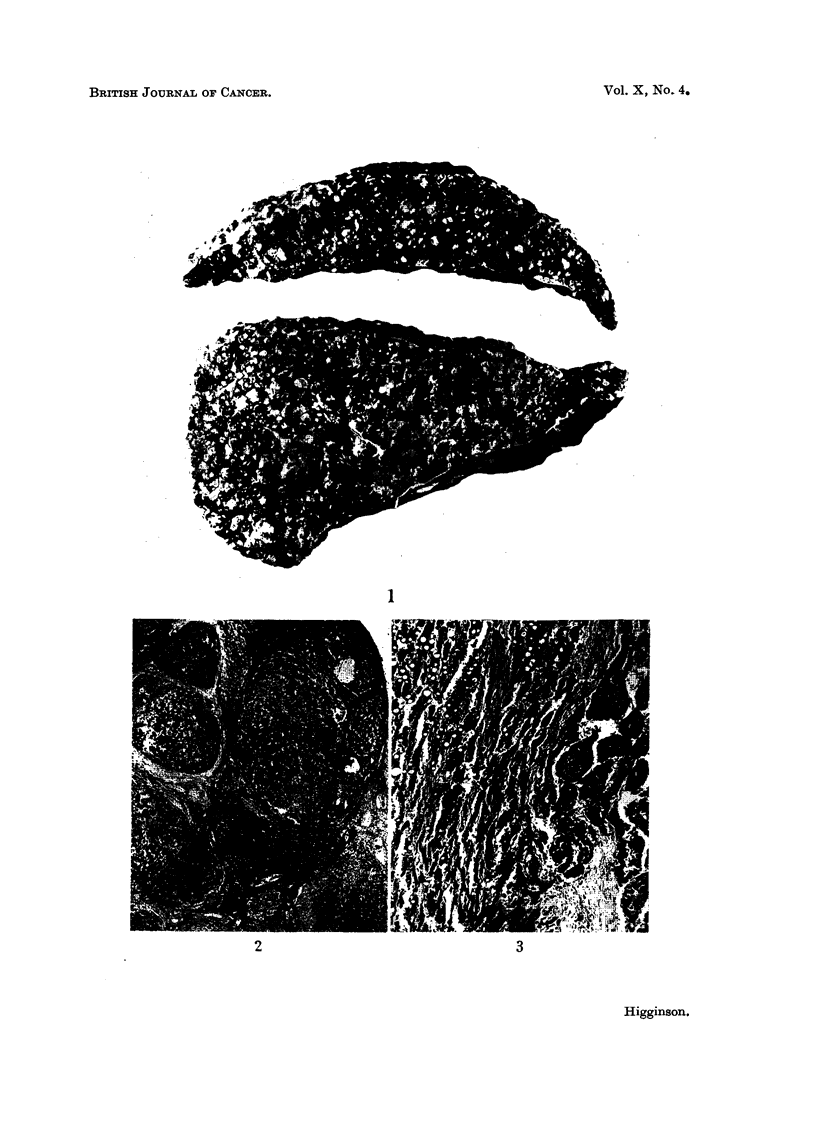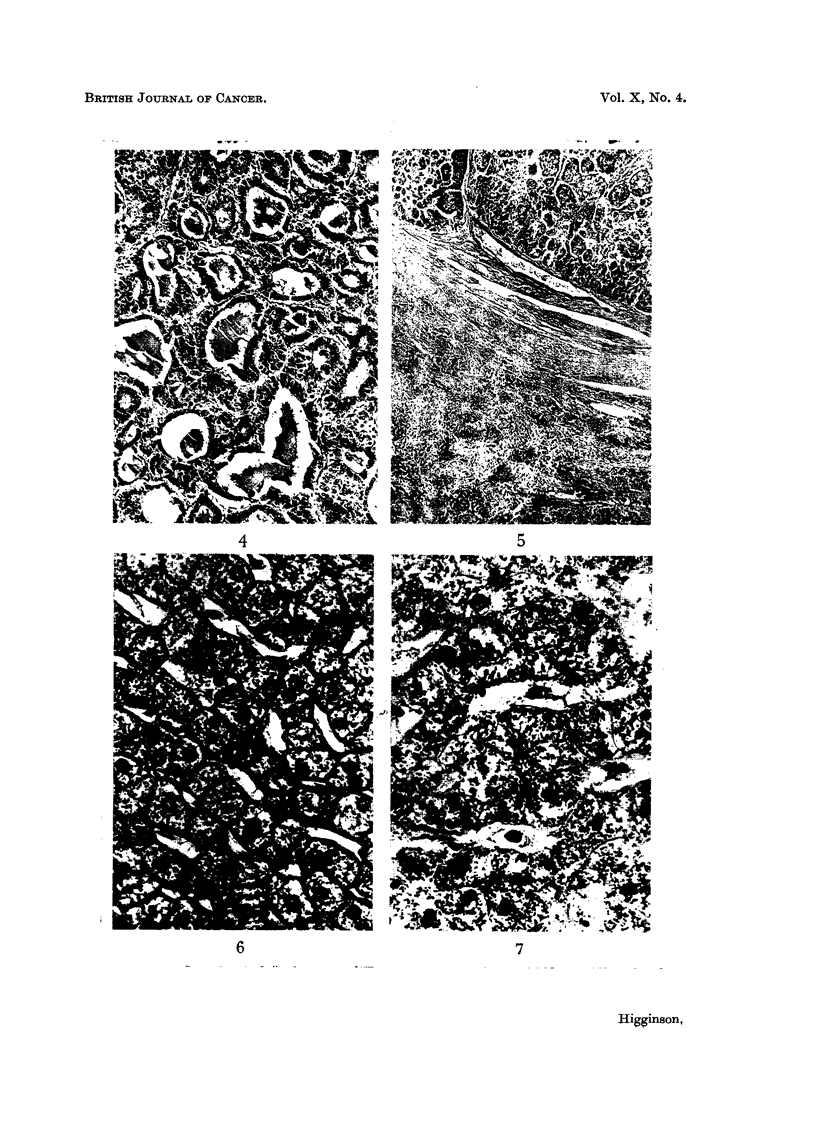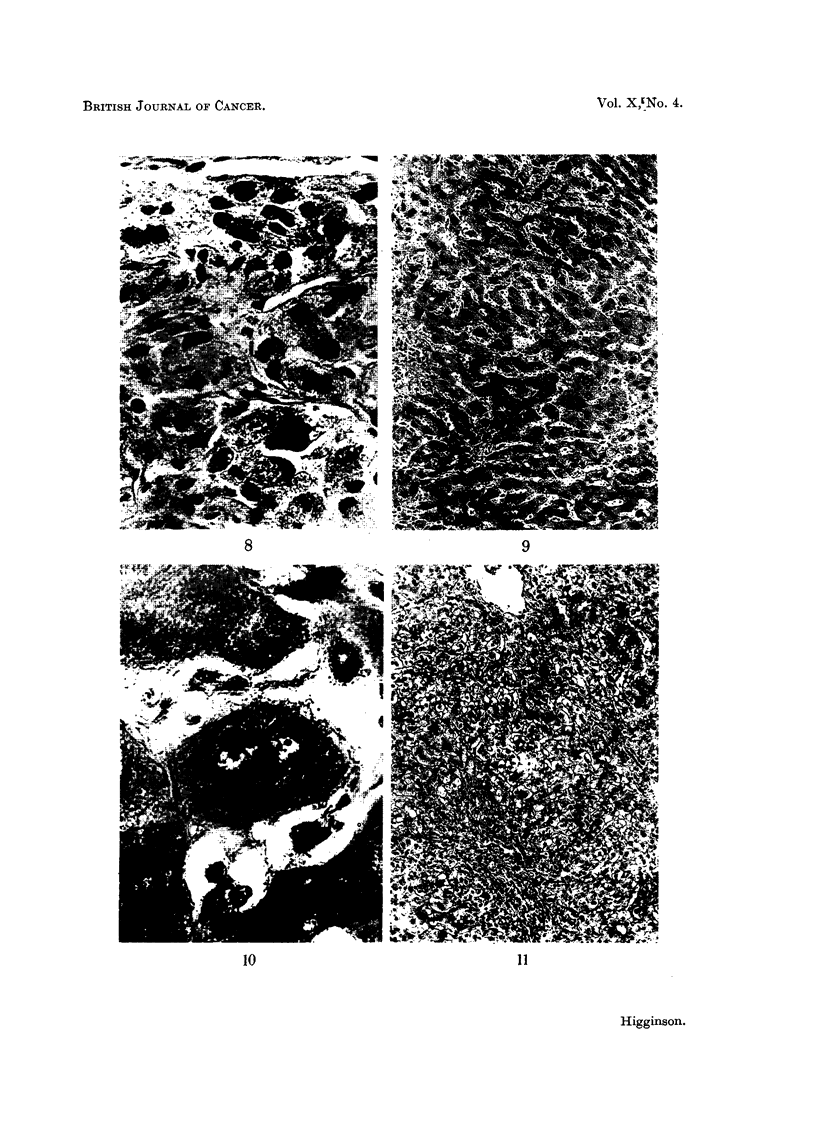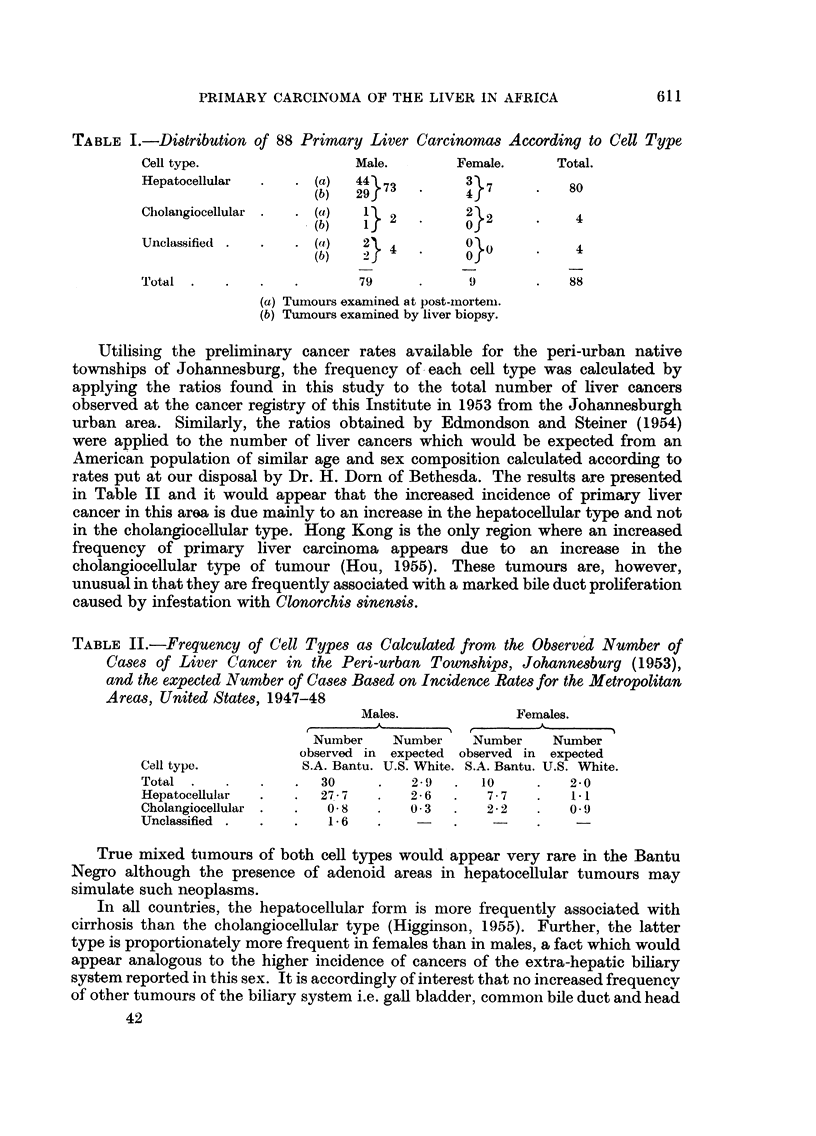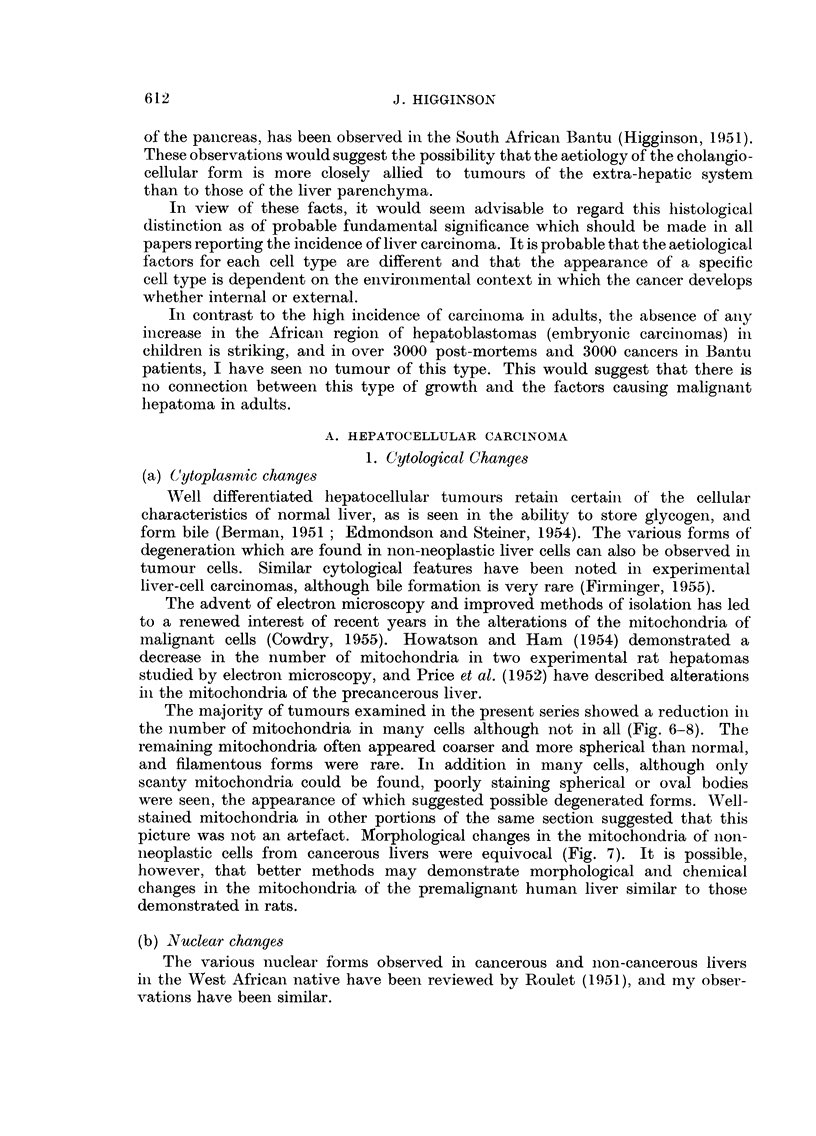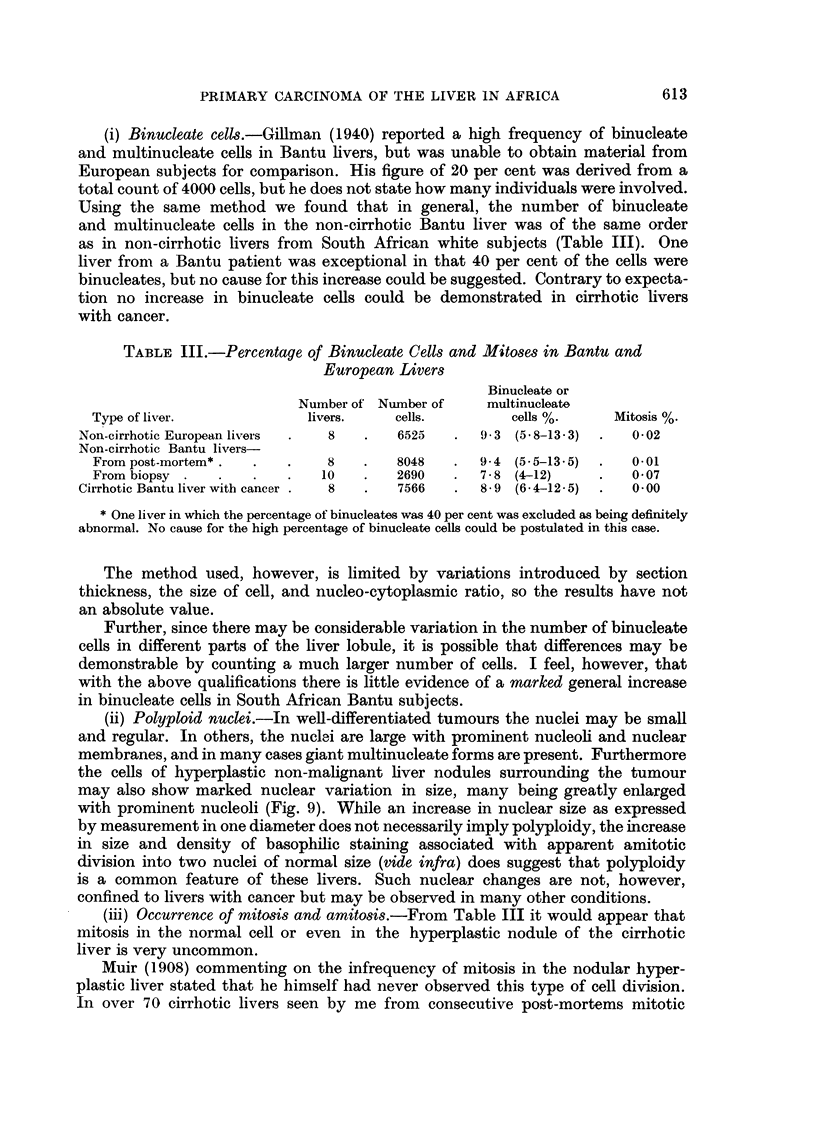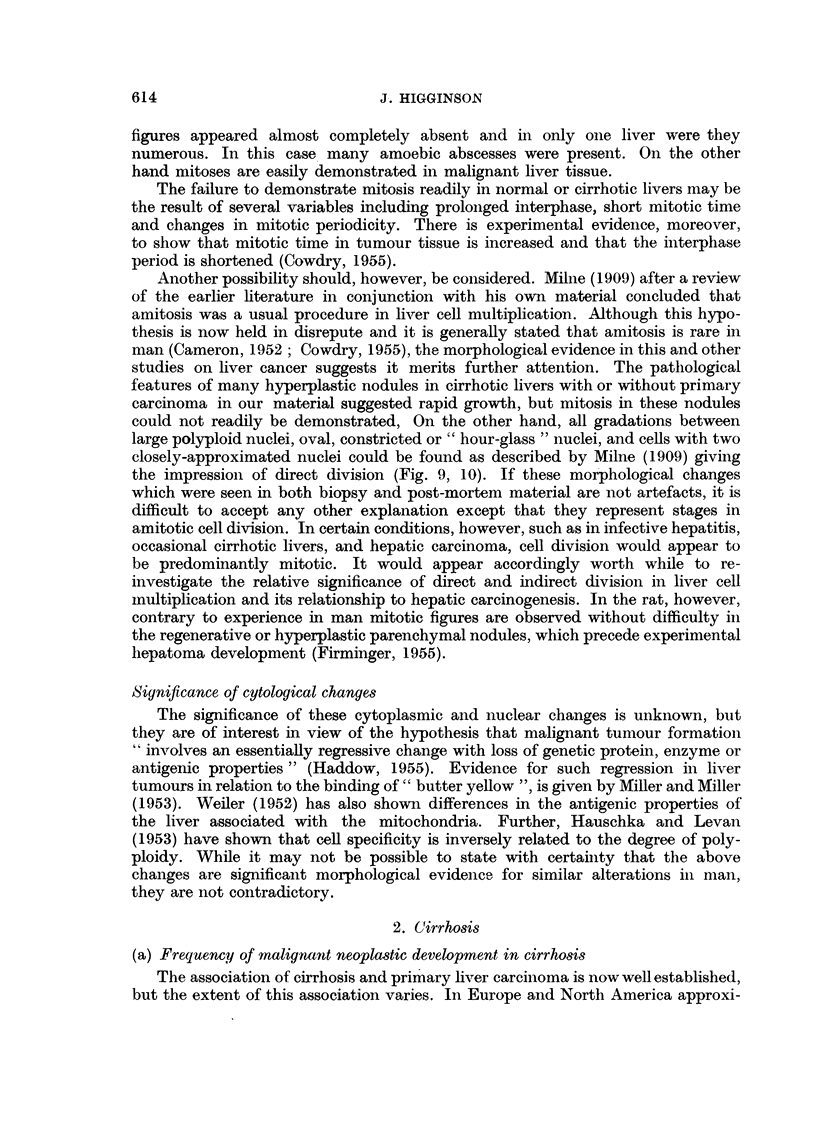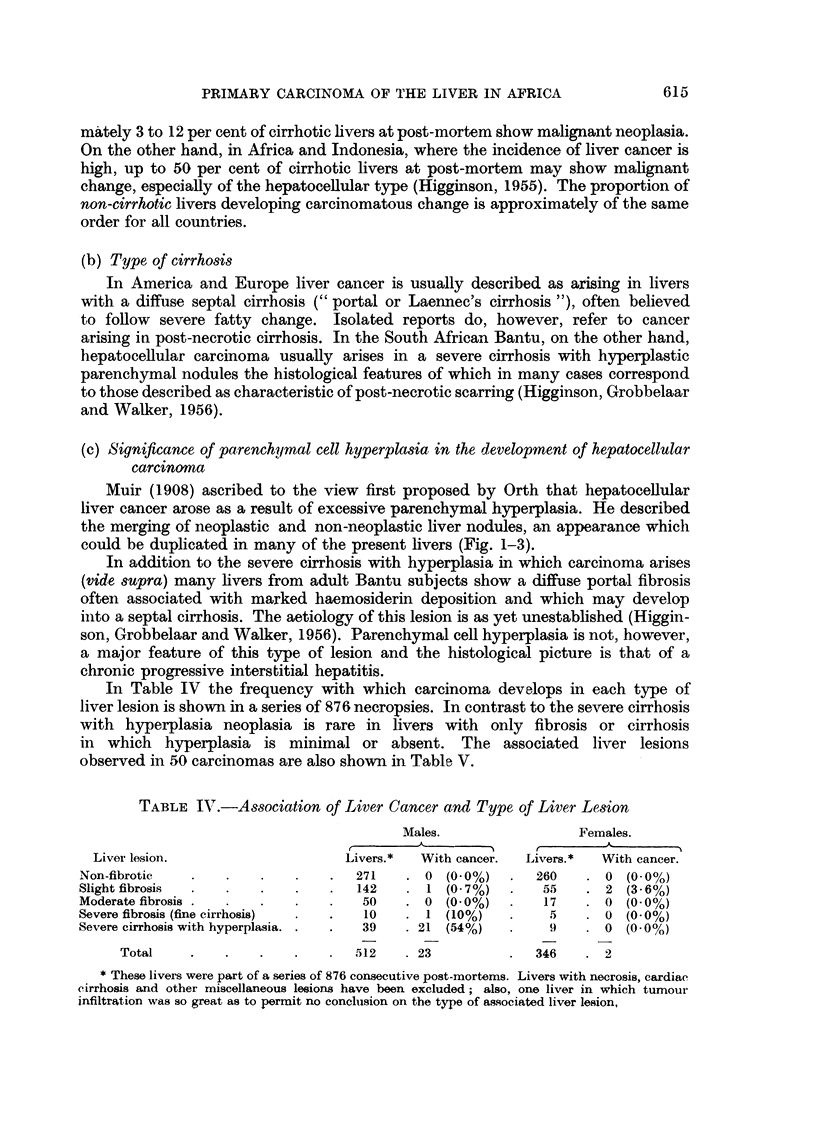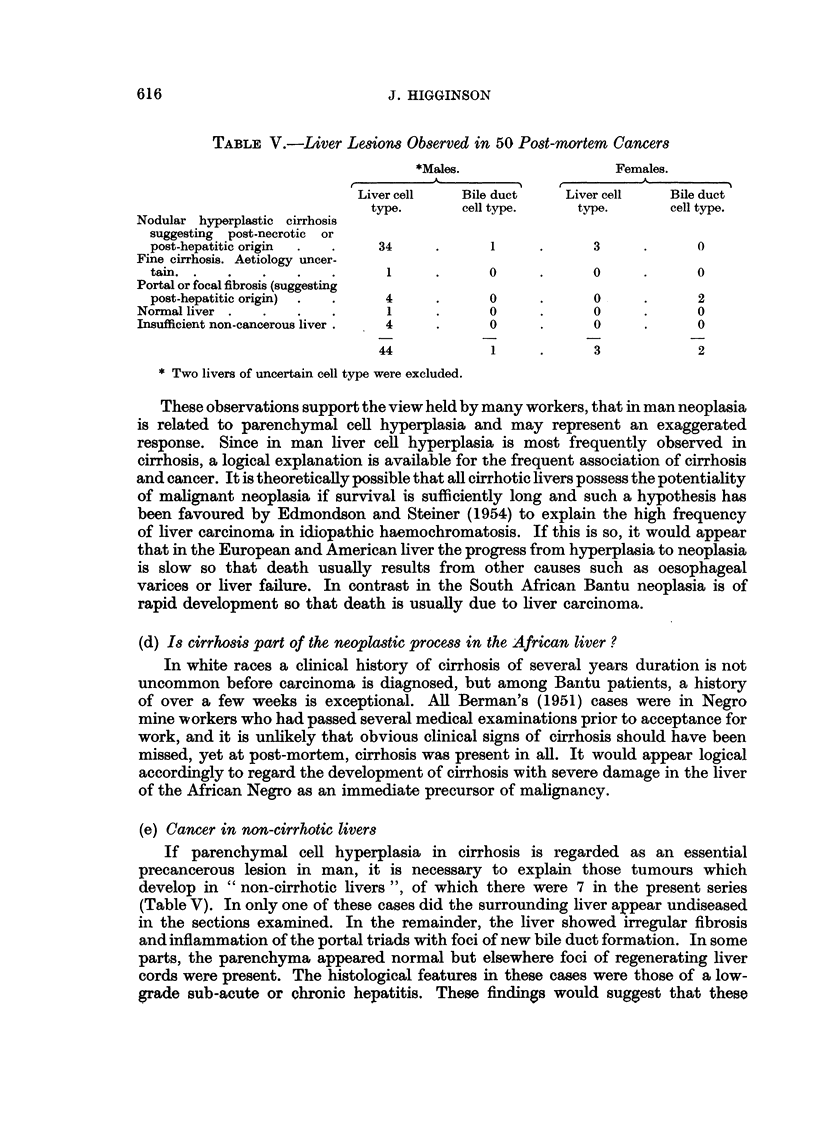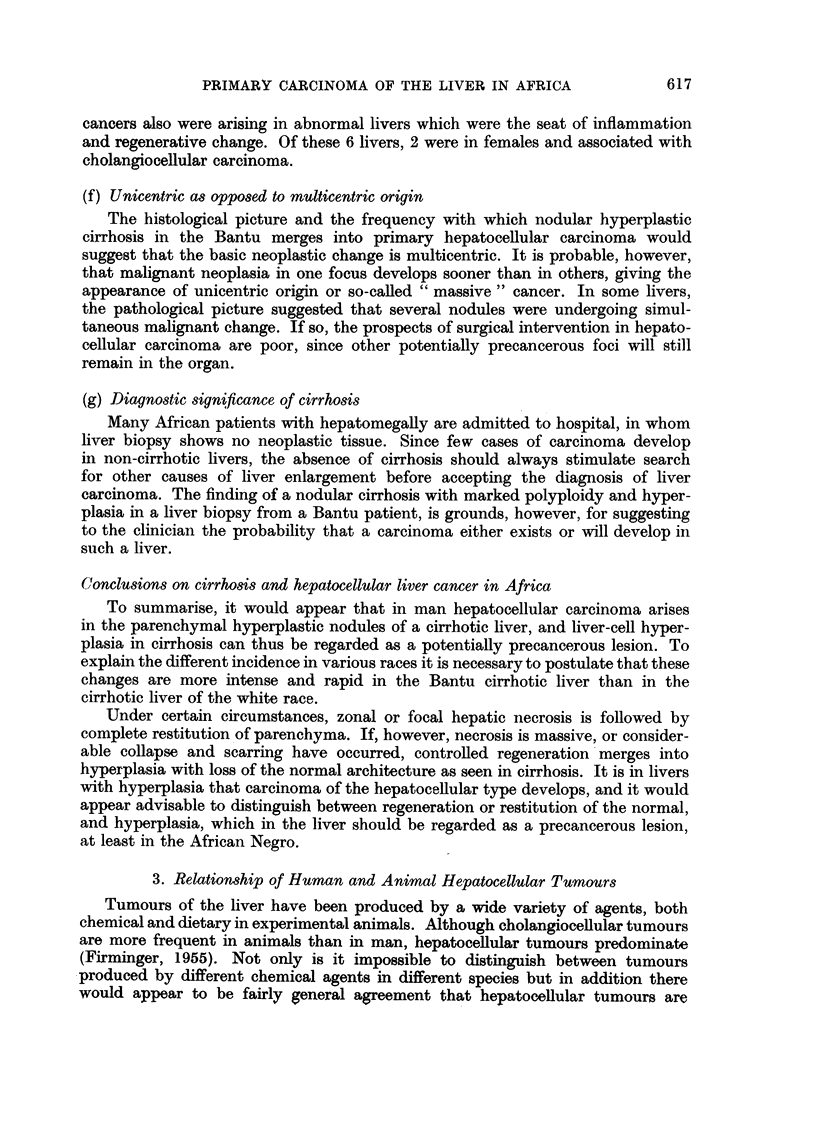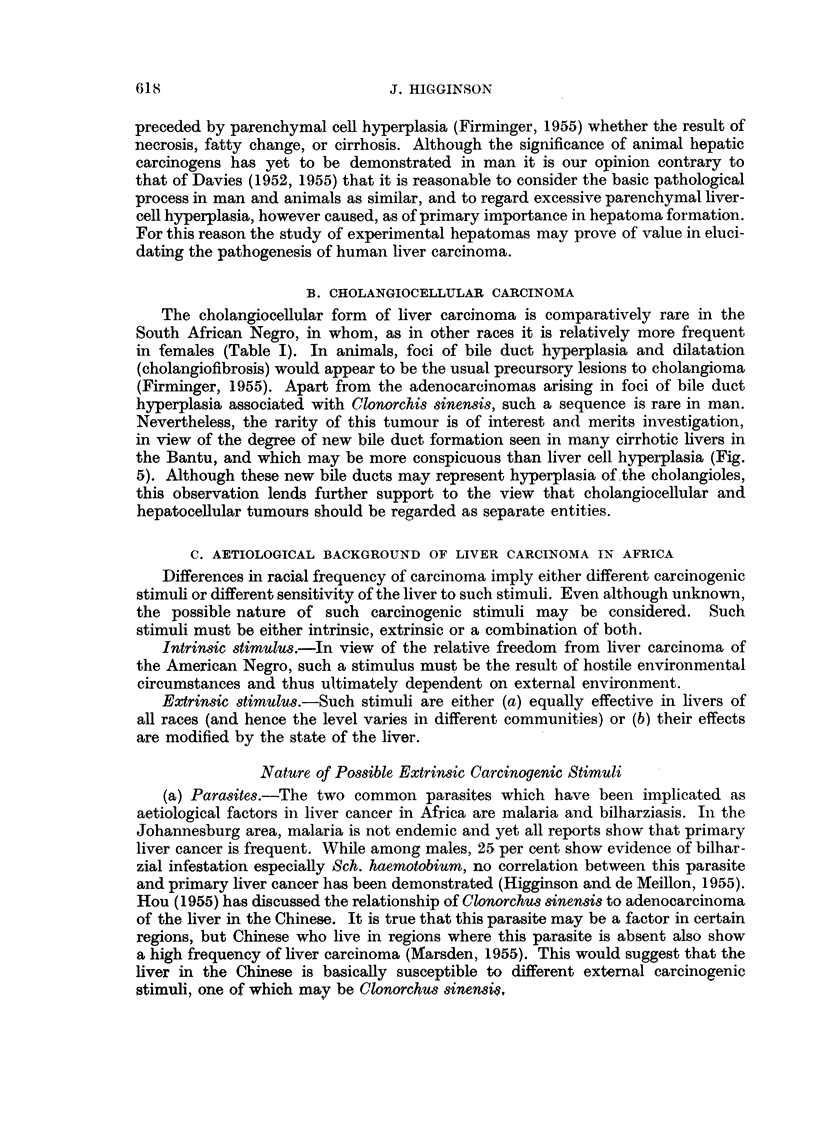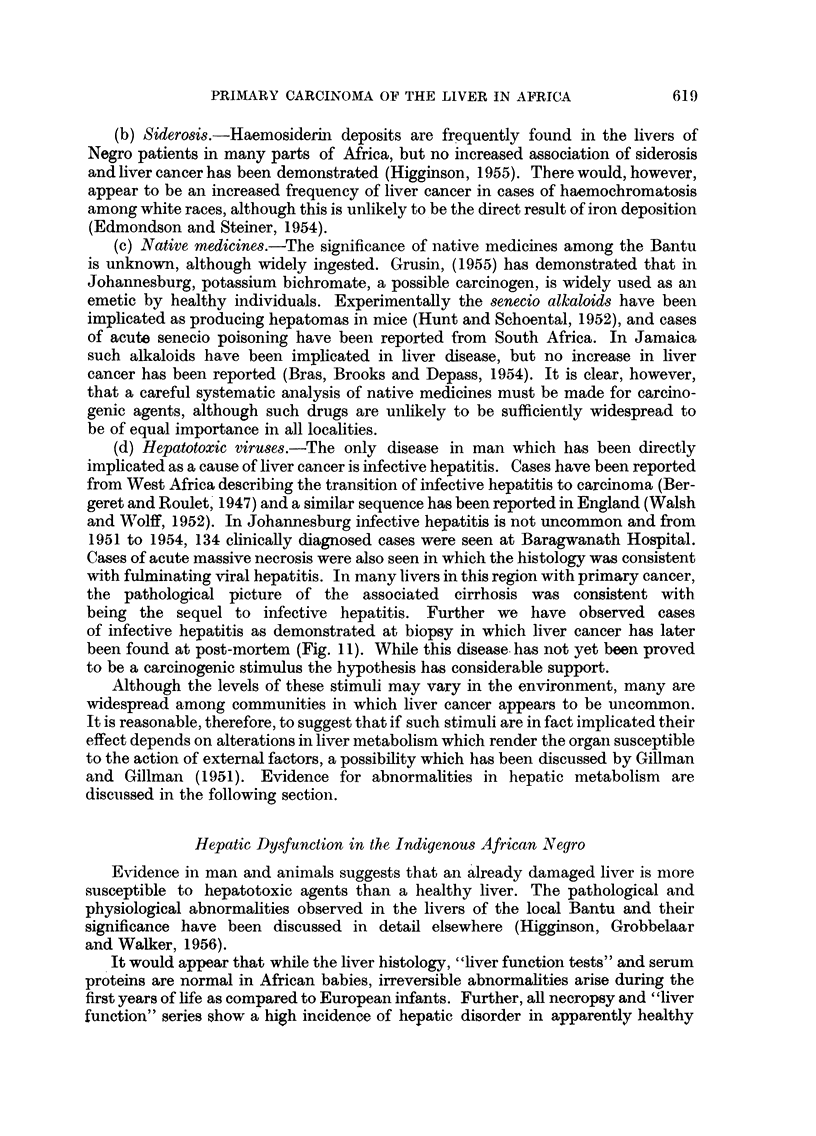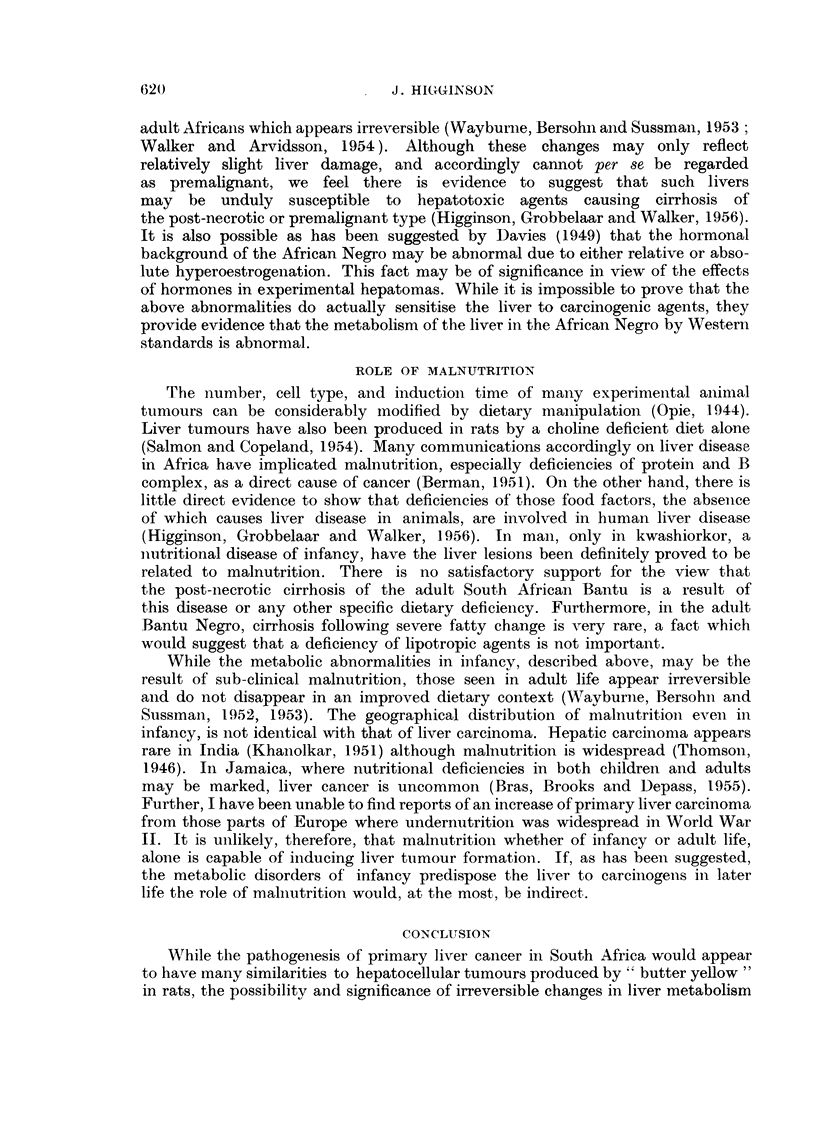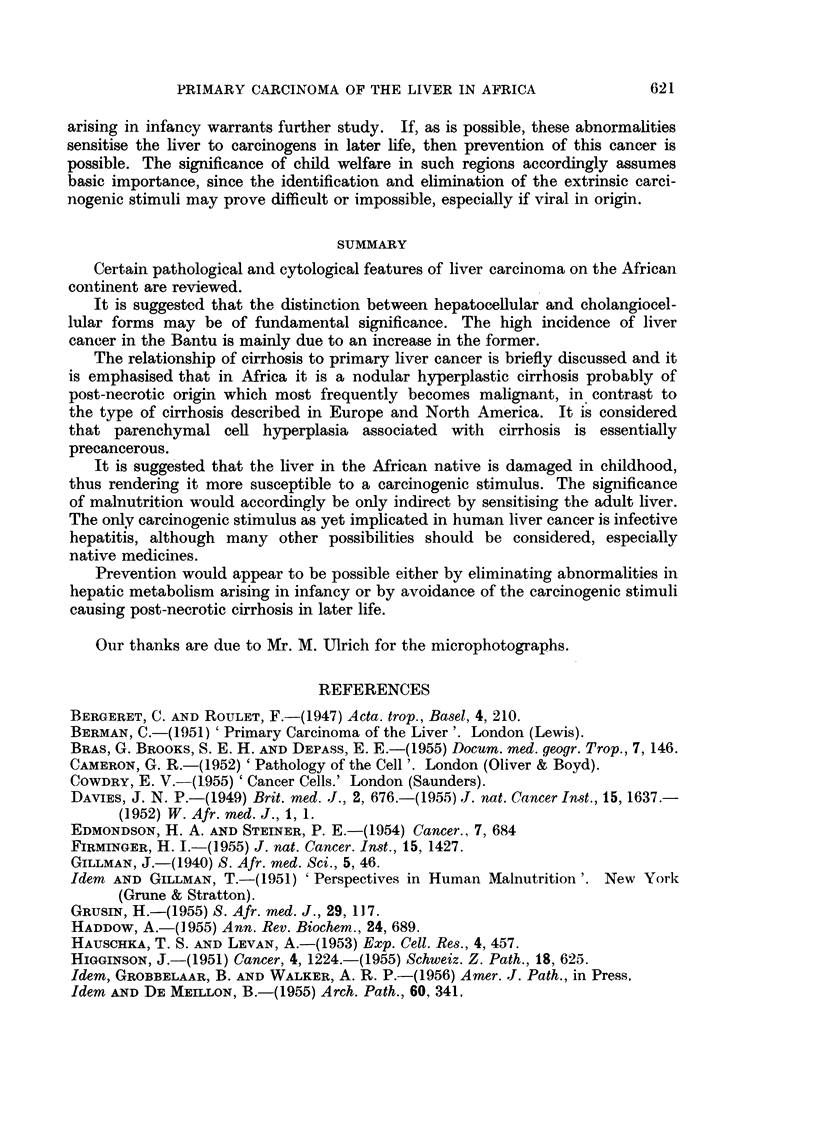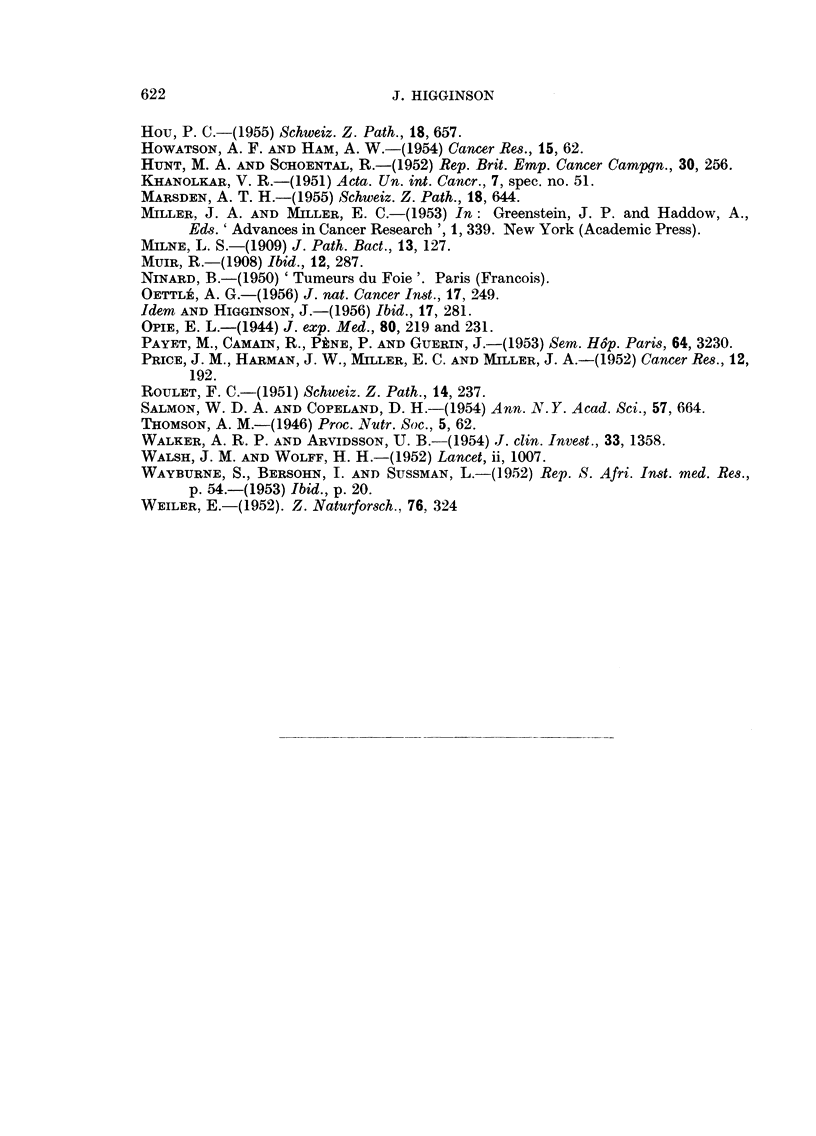# Primary Carcinoma of the Liver in Africa

**DOI:** 10.1038/bjc.1956.73

**Published:** 1956-12

**Authors:** J. Higginson

## Abstract

**Images:**


### PRIMARY CARCINOMA OF THE LIVER IN AFRICA

J. HIGGINSON

*From the South African Institute for Medical Research, Johannesburg, South Africa*

Received for publication September 8, 1956.

ALL reports reviewing the frequency of liver carcinoma in Africa, emphasise the high incidence of this neoplasm observed among the indigenous races (Ninard, 1950 ; Berman, 1951 ; Oettlé, 1956). From a study of liver cancer in the native townships around Johannesburg, it can be concluded that this increase is absolute and not due to a relative absence of other tumours (Oettlé and Higginson, 1956).

The present paper draws attention to the peculiar position occupied by the hepatocellular tumour in relation to this increase. The possible development and aetiological background of this type of growth is discussed in view of certain similarities to experimental animal hepatomas. Attention is also drawn to the view that despite its significance in experimental liver cancer, malnutrition is unlikely to be the only factor causing this high frequency in the African Negro.

### MATERIAL AND METHODS

This study is based on 53 primary carcinomas of the liver observed at necropsy at Baragwanath Hospital, Johannesburgh. Fifty-two tumours were from Bantu Negro subjects and one from a patient of Eurafrican descent. Surgical or needle biopsies were available in 15 of these, also in a further 36 carcinomas. In addition to the routine histological stains, mitochondria were demonstrated in liver biopsy material fixed in neutral formalin by Mallory's phosphotungstic haematoxylin after oxidation with potassium permanganate.

To estimate the proportion of multinucleate cells, sections were cut at 4 $\mu$ and groups of approximately 200–300 cells in contiguous fields were counted at 1250 magnification. Only cells with nuclei were included.

### GENERAL PATHOLOGICAL CONSIDERATIONS

The basic pathological picture of primary liver carcinoma would appear to be similar in all countries (Ninard, 1950 ; Berman, 1951 ; Edmondson and Steiner, 1954). Some pathological features of the tumours in the present series are illustrated in Fig. 1–5, and are identical to those previously described in this region (Berman, 1951). Certain characteristics, however, require amplification.

### *Significance of Tumour-cell Type*

Classically, liver tumours have been divided into hepatocellular and cholangiocellular types, but the significance of this classification has not yet been established. Berman (1951) states that " while the classification of primary liver cancer into hepatocellular and cholangiocellular carcinoma is useful for descriptive purposes, this division cannot be taken to mean that because a tumour assumes a particular pattern it is necessarily derived from the tissue it simulates " (p. 96). Ninard

(1950), however, regards hepatocellular tumours as specific and characteristic of the liver, and considers the cholangiocellular cell type non-specific and similar to any tumour of the intra- or extra-hepatic biliary system. On histogenic grounds, tumours of the liver cells and cholangioles should be regarded as " hepatocellular " or " liver cell type ", and tumours of the intra-hepatic bile ducts " cholangiocellular " or " bile duct type " ; but, while it would appear that the liver cell and bile duct anlages develop early and remain distinct, some workers believe cells of one type may develop into cells of the other.

Additional terminological confusion has been caused by difficulty in distinguishing the adenoid hepatocellular tumour from the cholangiocellular tumour (Fig. 4), and the former has sometimes been regarded as of the bile duct type. When present, we have regarded bile formation as pathognomic of the liver cell type.

In Table I the proportion of tumours of each cell type in the present series is shown, and is of the same order as that reported by Berman (1951) in mine workers, and in West African natives by Payet *et al.* (1953). The proportion of cholangiocellular tumours is lower, however, than that observed in Central Africa (Davies, 1952) and much lower than reported from America by Edmondson and Steiner (1954), although the latter authors took into account the adenoid hepatocellular type.

---

### EXPLANATION OF PLATES

FIG. 1.—Typical primary carcinoma in a liver showing a coarse nodular cirrhosis. Many of the parenchymal nodules in this specimen are undergoing malignant change. Zulu, male, aged 34 years.

FIG. 2.—Sections from same liver as Fig. 1 showing numerous foci of hepatocellular carcinoma. The surrounding liver shows foci of collapse and fibrosis suggesting post-necrotic scarring. Haematoxylin-eosin. × 20.

FIG. 3.—Section from a hepatocellular carcinoma showing merging of neoplastic and non-neoplastic trabeculae. Haematoxylin-eosin. × 75.

FIG. 4.—Section of an adenoid hepatocellular carcinoma. Although superficially resembling a cholangiocellular carcinoma these acini contain bile-stained material and the tumour cells can accordingly be regarded as bile producing, which places the neoplasm in the hepatocellular group. Haematoxylin-eosin. × 70.

FIG. 5.—Unusual case of hepatocellular carcinoma arising in a liver in which the parenchyma is almost completely destroyed and replaced by collapsed stroma with numerous newly-formed bile ducts. Despite the marked hyperplasia of the latter the tumour is hepatocellular. Haematoxylin-eosin. × 25.

FIG. 6.—Mitochondria in normal liver. Many appear grey in the photograph since they are slightly out of focus. P.A.H. × 450.

FIG. 7.—Mitochondria showing no significant change in cells of hyperplastic parenchymal nodule. Biopsy of this liver two months later demonstrated a typical hepatocellular carcinoma. P.A.H. × 450.

FIG. 8.—Hepatocellular carcinoma showing almost complete absence of mitochondria. The poorly-stained bodies in the cytoplasm suggest possible degenerated forms. P.A.H. × 450.

FIG. 9.—Numerous polyploid cells in liver nodule in region of hepatocellular tumour. Haematoxylin-eosin. × 90.

FIG. 10.—Large " hour-glass " nucleus in cell of hyperplastic liver nodule suggesting amitotic division. No spindle could be observed in this and other similar cells. Haematoxylin-eosin. × 750.

FIG. 11.—Liver biopsy from Bantu male, aged 21 years, showing the histological features of an infective hepatitis of some weeks duration. At post-mortem one month later a large hepatocellular carcinoma was present. Haematoxylin-eosin. × 70.

1

2

3

Higginson.

4

5

6

7

Higginson.

8

9

10

11

Higginson.

TABLE I.—*Distribution of* 88 *Primary Liver Carcinomas According to Cell Type*

| Cell type. | | Male. | | Female. | | Total. |
|---|---|---|---|---|---|---|
| Hepatocellular | (a) | 44 | 73 | 3 | 7 | 80 |
| | (b) | 29 | | 4 | | |
| Cholangiocellular | (a) | 1 | 2 | 2 | 2 | 4 |
| | (b) | 1 | | 0 | | |
| Unclassified | (a) | 2 | 4 | 0 | 0 | 4 |
| | (b) | 2 | | 0 | | |
| Total | | | 79 | | 9 | 88 |

(*a*) Tumours examined at post-mortem.
(*b*) Tumours examined by liver biopsy.

Utilising the preliminary cancer rates available for the peri-urban native townships of Johannesburg, the frequency of each cell type was calculated by applying the ratios found in this study to the total number of liver cancers observed at the cancer registry of this Institute in 1953 from the Johannesburgh urban area. Similarly, the ratios obtained by Edmondson and Steiner (1954) were applied to the number of liver cancers which would be expected from an American population of similar age and sex composition calculated according to rates put at our disposal by Dr. H. Dorn of Bethesda. The results are presented in Table II and it would appear that the increased incidence of primary liver cancer in this area is due mainly to an increase in the hepatocellular type and not in the cholangiocellular type. Hong Kong is the only region where an increased frequency of primary liver carcinoma appears due to an increase in the cholangiocellular type of tumour (Hou, 1955). These tumours are, however, unusual in that they are frequently associated with a marked bile duct proliferation caused by infestation with *Clonorchis sinensis.*

TABLE II.—*Frequency of Cell Types as Calculated from the Observed Number of Cases of Liver Cancer in the Peri-urban Townships, Johannesburg* (1953), *and the expected Number of Cases Based on Incidence Rates for the Metropolitan Areas, United States,* 1947–48

| | Males. | | Females. | |
|---|---|---|---|---|
| Cell type. | Number observed in S.A. Bantu. | Number expected U.S. White. | Number observed in S.A. Bantu. | Number expected U.S. White. |
| Total | 30 | 2·9 | 10 | 2·0 |
| Hepatocellular | 27·7 | 2·6 | 7·7 | 1·1 |
| Cholangiocellular | 0·8 | 0·3 | 2·2 | 0·9 |
| Unclassified | 1·6 | — | — | — |

True mixed tumours of both cell types would appear very rare in the Bantu Negro although the presence of adenoid areas in hepatocellular tumours may simulate such neoplasms.

In all countries, the hepatocellular form is more frequently associated with cirrhosis than the cholangiocellular type (Higginson, 1955). Further, the latter type is proportionately more frequent in females than in males, a fact which would appear analogous to the higher incidence of cancers of the extra-hepatic biliary system reported in this sex. It is accordingly of interest that no increased frequency of other tumours of the biliary system i.e. gall bladder, common bile duct and head of the pancreas, has been observed in the South African Bantu (Higginson, 1951). These observations would suggest the possibility that the aetiology of the cholangiocellular form is more closely allied to tumours of the extra-hepatic system than to those of the liver parenchyma.

In view of these facts, it would seem advisable to regard this histological distinction as of probable fundamental significance which should be made in all papers reporting the incidence of liver carcinoma. It is probable that the aetiological factors for each cell type are different and that the appearance of a specific cell type is dependent on the environmental context in which the cancer develops whether internal or external.

In contrast to the high incidence of carcinoma in adults, the absence of any increase in the African region of hepatoblastomas (embryonic carcinomas) in children is striking, and in over 3000 post-mortems and 3000 cancers in Bantu patients, I have seen no tumour of this type. This would suggest that there is no connection between this type of growth and the factors causing malignant hepatoma in adults.

### A. HEPATOCELLULAR CARCINOMA

### 1. *Cytological Changes*

(a) *Cytoplasmic changes*

Well differentiated hepatocellular tumours retain certain of the cellular characteristics of normal liver, as is seen in the ability to store glycogen, and form bile (Berman, 1951 ; Edmondson and Steiner, 1954). The various forms of degeneration which are found in non-neoplastic liver cells can also be observed in tumour cells. Similar cytological features have been noted in experimental liver-cell carcinomas, although bile formation is very rare (Firminger, 1955).

The advent of electron microscopy and improved methods of isolation has led to a renewed interest of recent years in the alterations of the mitochondria of malignant cells (Cowdry, 1955). Howatson and Ham (1954) demonstrated a decrease in the number of mitochondria in two experimental rat hepatomas studied by electron microscopy, and Price *et al.* (1952) have described alterations in the mitochondria of the precancerous liver.

The majority of tumours examined in the present series showed a reduction in the number of mitochondria in many cells although not in all (Fig. 6–8). The remaining mitochondria often appeared coarser and more spherical than normal, and filamentous forms were rare. In addition in many cells, although only scanty mitochondria could be found, poorly staining spherical or oval bodies were seen, the appearance of which suggested possible degenerated forms. Well-stained mitochondria in other portions of the same section suggested that this picture was not an artefact. Morphological changes in the mitochondria of non-neoplastic cells from cancerous livers were equivocal (Fig. 7). It is possible, however, that better methods may demonstrate morphological and chemical changes in the mitochondria of the premalignant human liver similar to those demonstrated in rats.

(b) *Nuclear changes*

The various nuclear forms observed in cancerous and non-cancerous livers in the West African native have been reviewed by Roulet (1951), and my observations have been similar.

(i) *Binucleate cells.*—Gillman (1940) reported a high frequency of binucleate and multinucleate cells in Bantu livers, but was unable to obtain material from European subjects for comparison. His figure of 20 per cent was derived from a total count of 4000 cells, but he does not state how many individuals were involved. Using the same method we found that in general, the number of binucleate and multinucleate cells in the non-cirrhotic Bantu liver was of the same order as in non-cirrhotic livers from South African white subjects (Table III). One liver from a Bantu patient was exceptional in that 40 per cent of the cells were binucleates, but no cause for this increase could be suggested. Contrary to expectation no increase in binucleate cells could be demonstrated in cirrhotic livers with cancer.

TABLE III.—*Percentage of Binucleate Cells and Mitoses in Bantu and European Livers*

| Type of liver. | Number of livers. | Number of cells. | Binucleate or multinucleate cells %. | Mitosis %. |
|---|---|---|---|---|
| Non-cirrhotic European livers | 8 | 6525 | 9·3 (5·8–13·3) | 0·02 |
| Non-cirrhotic Bantu livers— | | | | |
| From post-mortem* | 8 | 8048 | 9·4 (5·5–13·5) | 0·01 |
| From biopsy | 10 | 2690 | 7·8 (4–12) | 0·07 |
| Cirrhotic Bantu liver with cancer | 8 | 7566 | 8·9 (6·4–12·5) | 0·00 |

* One liver in which the percentage of binucleates was 40 per cent was excluded as being definitely abnormal. No cause for the high percentage of binucleate cells could be postulated in this case.

The method used, however, is limited by variations introduced by section thickness, the size of cell, and nucleo-cytoplasmic ratio, so the results have not an absolute value.

Further, since there may be considerable variation in the number of binucleate cells in different parts of the liver lobule, it is possible that differences may be demonstrable by counting a much larger number of cells. I feel, however, that with the above qualifications there is little evidence of a *marked* general increase in binucleate cells in South African Bantu subjects.

(ii) *Polyploid nuclei.*—In well-differentiated tumours the nuclei may be small and regular. In others, the nuclei are large with prominent nucleoli and nuclear membranes, and in many cases giant multinucleate forms are present. Furthermore the cells of hyperplastic non-malignant liver nodules surrounding the tumour may also show marked nuclear variation in size, many being greatly enlarged with prominent nucleoli (Fig. 9). While an increase in nuclear size as expressed by measurement in one diameter does not necessarily imply polyploidy, the increase in size and density of basophilic staining associated with apparent amitotic division into two nuclei of normal size (*vide infra*) does suggest that polyploidy is a common feature of these livers. Such nuclear changes are not, however, confined to livers with cancer but may be observed in many other conditions.

(iii) *Occurrence of mitosis and amitosis.*—From Table III it would appear that mitosis in the normal cell or even in the hyperplastic nodule of the cirrhotic liver is very uncommon.

Muir (1908) commenting on the infrequency of mitosis in the nodular hyperplastic liver stated that he himself had never observed this type of cell division. In over 70 cirrhotic livers seen by me from consecutive post-mortems mitotic figures appeared almost completely absent and in only one liver were they numerous. In this case many amoebic abscesses were present. On the other hand mitoses are easily demonstrated in malignant liver tissue.

The failure to demonstrate mitosis readily in normal or cirrhotic livers may be the result of several variables including prolonged interphase, short mitotic time and changes in mitotic periodicity. There is experimental evidence, moreover, to show that mitotic time in tumour tissue is increased and that the interphase period is shortened (Cowdry, 1955).

Another possibility should, however, be considered. Milne (1909) after a review of the earlier literature in conjunction with his own material concluded that amitosis was a usual procedure in liver cell multiplication. Although this hypothesis is now held in disrepute and it is generally stated that amitosis is rare in man (Cameron, 1952 ; Cowdry, 1955), the morphological evidence in this and other studies on liver cancer suggests it merits further attention. The pathological features of many hyperplastic nodules in cirrhotic livers with or without primary carcinoma in our material suggested rapid growth, but mitosis in these nodules could not readily be demonstrated, On the other hand, all gradations between large polyploid nuclei, oval, constricted or " hour-glass " nuclei, and cells with two closely-approximated nuclei could be found as described by Milne (1909) giving the impression of direct division (Fig. 9, 10). If these morphological changes which were seen in both biopsy and post-mortem material are not artefacts, it is difficult to accept any other explanation except that they represent stages in amitotic cell division. In certain conditions, however, such as in infective hepatitis, occasional cirrhotic livers, and hepatic carcinoma, cell division would appear to be predominantly mitotic. It would appear accordingly worth while to re-investigate the relative significance of direct and indirect division in liver cell multiplication and its relationship to hepatic carcinogenesis. In the rat, however, contrary to experience in man mitotic figures are observed without difficulty in the regenerative or hyperplastic parenchymal nodules, which precede experimental hepatoma development (Firminger, 1955).

*Significance of cytological changes*

The significance of these cytoplasmic and nuclear changes is unknown, but they are of interest in view of the hypothesis that malignant tumour formation " involves an essentially regressive change with loss of genetic protein, enzyme or antigenic properties " (Haddow, 1955). Evidence for such regression in liver tumours in relation to the binding of " butter yellow ", is given by Miller and Miller (1953). Weiler (1952) has also shown differences in the antigenic properties of the liver associated with the mitochondria. Further, Hauschka and Levan (1953) have shown that cell specificity is inversely related to the degree of polyploidy. While it may not be possible to state with certainty that the above changes are significant morphological evidence for similar alterations in man, they are not contradictory.

### 2. *Cirrhosis*

### (a) *Frequency of malignant neoplastic development in cirrhosis*

The association of cirrhosis and primary liver carcinoma is now well established, but the extent of this association varies. In Europe and North America approximately 3 to 12 per cent of cirrhotic livers at post-mortem show malignant neoplasia. On the other hand, in Africa and Indonesia, where the incidence of liver cancer is high, up to 50 per cent of cirrhotic livers at post-mortem may show malignant change, especially of the hepatocellular type (Higginson, 1955). The proportion of *non-cirrhotic* livers developing carcinomatous change is approximately of the same order for all countries.

(b) *Type of cirrhosis*

In America and Europe liver cancer is usually described as arising in livers with a diffuse septal cirrhosis ("portal or Laennec's cirrhosis"), often believed to follow severe fatty change. Isolated reports do, however, refer to cancer arising in post-necrotic cirrhosis. In the South African Bantu, on the other hand, hepatocellular carcinoma usually arises in a severe cirrhosis with hyperplastic parenchymal nodules the histological features of which in many cases correspond to those described as characteristic of post-necrotic scarring (Higginson, Grobbelaar and Walker, 1956).

(c) *Significance of parenchymal cell hyperplasia in the development of hepatocellular carcinoma*

Muir (1908) ascribed to the view first proposed by Orth that hepatocellular liver cancer arose as a result of excessive parenchymal hyperplasia. He described the merging of neoplastic and non-neoplastic liver nodules, an appearance which could be duplicated in many of the present livers (Fig. 1–3).

In addition to the severe cirrhosis with hyperplasia in which carcinoma arises (*vide supra*) many livers from adult Bantu subjects show a diffuse portal fibrosis often associated with marked haemosiderin deposition and which may develop into a septal cirrhosis. The aetiology of this lesion is as yet unestablished (Higginson, Grobbelaar and Walker, 1956). Parenchymal cell hyperplasia is not, however, a major feature of this type of lesion and the histological picture is that of a chronic progressive interstitial hepatitis.

In Table IV the frequency with which carcinoma develops in each type of liver lesion is shown in a series of 876 necropsies. In contrast to the severe cirrhosis with hyperplasia neoplasia is rare in livers with only fibrosis or cirrhosis in which hyperplasia is minimal or absent. The associated liver lesions observed in 50 carcinomas are also shown in Table V.

TABLE IV.—*Association of Liver Cancer and Type of Liver Lesion*

| | Males. | | Females. | |
|---|---|---|---|---|
| Liver lesion. | Livers.* | With cancer. | Livers.* | With cancer. |
| Non-fibrotic | 271 | 0 (0·0%) | 260 | 0 (0·0%) |
| Slight fibrosis | 142 | 1 (0·7%) | 55 | 2 (3·6%) |
| Moderate fibrosis | 50 | 0 (0·0%) | 17 | 0 (0·0%) |
| Severe fibrosis (fine cirrhosis) | 10 | 1 (10%) | 5 | 0 (0·0%) |
| Severe cirrhosis with hyperplasia | 39 | 21 (54%) | 9 | 0 (0·0%) |
| Total | 512 | 23 | 346 | 2 |

* These livers were part of a series of 876 consecutive post-mortems. Livers with necrosis, cardiac cirrhosis and other miscellaneous lesions have been excluded; also, one liver in which tumour infiltration was so great as to permit no conclusion on the type of associated liver lesion.

TABLE V.—*Liver Lesions Observed in 50 Post-mortem Cancers*

| | *Males. | | Females. | |
|---|---|---|---|---|
| | Liver cell type. | Bile duct cell type. | Liver cell type. | Bile duct cell type. |
| Nodular hyperplastic cirrhosis suggesting post-necrotic or post-hepatitic origin | 34 | 1 | 3 | 0 |
| Fine cirrhosis. Aetiology uncertain. | 1 | 0 | 0 | 0 |
| Portal or focal fibrosis (suggesting post-hepatitic origin) | 4 | 0 | 0 | 2 |
| Normal liver | 1 | 0 | 0 | 0 |
| Insufficient non-cancerous liver | 4 | 0 | 0 | 0 |
| | 44 | 1 | 3 | 2 |

* Two livers of uncertain cell type were excluded.

These observations support the view held by many workers, that in man neoplasia is related to parenchymal cell hyperplasia and may represent an exaggerated response. Since in man liver cell hyperplasia is most frequently observed in cirrhosis, a logical explanation is available for the frequent association of cirrhosis and cancer. It is theoretically possible that all cirrhotic livers possess the potentiality of malignant neoplasia if survival is sufficiently long and such a hypothesis has been favoured by Edmondson and Steiner (1954) to explain the high frequency of liver carcinoma in idiopathic haemochromatosis. If this is so, it would appear that in the European and American liver the progress from hyperplasia to neoplasia is slow so that death usually results from other causes such as oesophageal varices or liver failure. In contrast in the South African Bantu neoplasia is of rapid development so that death is usually due to liver carcinoma.

(d) *Is cirrhosis part of the neoplastic process in the African liver ?*

In white races a clinical history of cirrhosis of several years duration is not uncommon before carcinoma is diagnosed, but among Bantu patients, a history of over a few weeks is exceptional. All Berman's (1951) cases were in Negro mine workers who had passed several medical examinations prior to acceptance for work, and it is unlikely that obvious clinical signs of cirrhosis should have been missed, yet at post-mortem, cirrhosis was present in all. It would appear logical accordingly to regard the development of cirrhosis with severe damage in the liver of the African Negro as an immediate precursor of malignancy.

(e) *Cancer in non-cirrhotic livers*

If parenchymal cell hyperplasia in cirrhosis is regarded as an essential precancerous lesion in man, it is necessary to explain those tumours which develop in " non-cirrhotic livers ", of which there were 7 in the present series (Table V). In only one of these cases did the surrounding liver appear undiseased in the sections examined. In the remainder, the liver showed irregular fibrosis and inflammation of the portal triads with foci of new bile duct formation. In some parts, the parenchyma appeared normal but elsewhere foci of regenerating liver cords were present. The histological features in these cases were those of a low-grade sub-acute or chronic hepatitis. These findings would suggest that these cancers also were arising in abnormal livers which were the seat of inflammation and regenerative change. Of these 6 livers, 2 were in females and associated with cholangiocellular carcinoma.

(f) *Unicentric as opposed to multicentric origin*

The histological picture and the frequency with which nodular hyperplastic cirrhosis in the Bantu merges into primary hepatocellular carcinoma would suggest that the basic neoplastic change is multicentric. It is probable, however, that malignant neoplasia in one focus develops sooner than in others, giving the appearance of unicentric origin or so-called " massive " cancer. In some livers, the pathological picture suggested that several nodules were undergoing simultaneous malignant change. If so, the prospects of surgical intervention in hepatocellular carcinoma are poor, since other potentially precancerous foci will still remain in the organ.

(g) *Diagnostic significance of cirrhosis*

Many African patients with hepatomegally are admitted to hospital, in whom liver biopsy shows no neoplastic tissue. Since few cases of carcinoma develop in non-cirrhotic livers, the absence of cirrhosis should always stimulate search for other causes of liver enlargement before accepting the diagnosis of liver carcinoma. The finding of a nodular cirrhosis with marked polyploidy and hyperplasia in a liver biopsy from a Bantu patient, is grounds, however, for suggesting to the clinician the probability that a carcinoma either exists or will develop in such a liver.

*Conclusions on cirrhosis and hepatocellular liver cancer in Africa*

To summarise, it would appear that in man hepatocellular carcinoma arises in the parenchymal hyperplastic nodules of a cirrhotic liver, and liver-cell hyperplasia in cirrhosis can thus be regarded as a potentially precancerous lesion. To explain the different incidence in various races it is necessary to postulate that these changes are more intense and rapid in the Bantu cirrhotic liver than in the cirrhotic liver of the white race.

Under certain circumstances, zonal or focal hepatic necrosis is followed by complete restitution of parenchyma. If, however, necrosis is massive, or considerable collapse and scarring have occurred, controlled regeneration merges into hyperplasia with loss of the normal architecture as seen in cirrhosis. It is in livers with hyperplasia that carcinoma of the hepatocellular type develops, and it would appear advisable to distinguish between regeneration or restitution of the normal, and hyperplasia, which in the liver should be regarded as a precancerous lesion, at least in the African Negro.

### 3. *Relationship of Human and Animal Hepatocellular Tumours*

Tumours of the liver have been produced by a wide variety of agents, both chemical and dietary in experimental animals. Although cholangiocellular tumours are more frequent in animals than in man, hepatocellular tumours predominate (Firminger, 1955). Not only is it impossible to distinguish between tumours produced by different chemical agents in different species but in addition there would appear to be fairly general agreement that hepatocellular tumours are preceded by parenchymal cell hyperplasia (Firminger, 1955) whether the result of necrosis, fatty change, or cirrhosis. Although the significance of animal hepatic carcinogens has yet to be demonstrated in man it is our opinion contrary to that of Davies (1952, 1955) that it is reasonable to consider the basic pathological process in man and animals as similar, and to regard excessive parenchymal liver-cell hyperplasia, however caused, as of primary importance in hepatoma formation. For this reason the study of experimental hepatomas may prove of value in elucidating the pathogenesis of human liver carcinoma.

### B. CHOLANGIOCELLULAR CARCINOMA

The cholangiocellular form of liver carcinoma is comparatively rare in the South African Negro, in whom, as in other races it is relatively more frequent in females (Table I). In animals, foci of bile duct hyperplasia and dilatation (cholangiofibrosis) would appear to be the usual precursory lesions to cholangioma (Firminger, 1955). Apart from the adenocarcinomas arising in foci of bile duct hyperplasia associated with *Clonorchis sinensis*, such a sequence is rare in man. Nevertheless, the rarity of this tumour is of interest and merits investigation, in view of the degree of new bile duct formation seen in many cirrhotic livers in the Bantu, and which may be more conspicuous than liver cell hyperplasia (Fig. 5). Although these new bile ducts may represent hyperplasia of the cholangioles, this observation lends further support to the view that cholangiocellular and hepatocellular tumours should be regarded as separate entities.

### C. AETIOLOGICAL BACKGROUND OF LIVER CARCINOMA IN AFRICA

Differences in racial frequency of carcinoma imply either different carcinogenic stimuli or different sensitivity of the liver to such stimuli. Even although unknown, the possible nature of such carcinogenic stimuli may be considered. Such stimuli must be either intrinsic, extrinsic or a combination of both.

*Intrinsic stimulus.*—In view of the relative freedom from liver carcinoma of the American Negro, such a stimulus must be the result of hostile environmental circumstances and thus ultimately dependent on external environment.

*Extrinsic stimulus.*—Such stimuli are either (*a*) equally effective in livers of all races (and hence the level varies in different communities) or (*b*) their effects are modified by the state of the liver.

### *Nature of Possible Extrinsic Carcinogenic Stimuli*

(a) *Parasites.*—The two common parasites which have been implicated as aetiological factors in liver cancer in Africa are malaria and bilharziasis. In the Johannesburg area, malaria is not endemic and yet all reports show that primary liver cancer is frequent. While among males, 25 per cent show evidence of bilharzial infestation especially *Sch. haemotobium*, no correlation between this parasite and primary liver cancer has been demonstrated (Higginson and de Meillon, 1955). Hou (1955) has discussed the relationship of *Clonorchus sinensis* to adenocarcinoma of the liver in the Chinese. It is true that this parasite may be a factor in certain regions, but Chinese who live in regions where this parasite is absent also show a high frequency of liver carcinoma (Marsden, 1955). This would suggest that the liver in the Chinese is basically susceptible to different external carcinogenic stimuli, one of which may be *Clonorchus sinensis*.

(b) *Siderosis.*—Haemosiderin deposits are frequently found in the livers of Negro patients in many parts of Africa, but no increased association of siderosis and liver cancer has been demonstrated (Higginson, 1955). There would, however, appear to be an increased frequency of liver cancer in cases of haemochromatosis among white races, although this is unlikely to be the direct result of iron deposition (Edmondson and Steiner, 1954).

(c) *Native medicines.*—The significance of native medicines among the Bantu is unknown, although widely ingested. Grusin, (1955) has demonstrated that in Johannesburg, potassium bichromate, a possible carcinogen, is widely used as an emetic by healthy individuals. Experimentally the *senecio alkaloids* have been implicated as producing hepatomas in mice (Hunt and Schoental, 1952), and cases of acute senecio poisoning have been reported from South Africa. In Jamaica such alkaloids have been implicated in liver disease, but no increase in liver cancer has been reported (Bras, Brooks and Depass, 1954). It is clear, however, that a careful systematic analysis of native medicines must be made for carcinogenic agents, although such drugs are unlikely to be sufficiently widespread to be of equal importance in all localities.

(d) *Hepatotoxic viruses.*—The only disease in man which has been directly implicated as a cause of liver cancer is infective hepatitis. Cases have been reported from West Africa describing the transition of infective hepatitis to carcinoma (Bergeret and Roulet, 1947) and a similar sequence has been reported in England (Walsh and Wolff, 1952). In Johannesburg infective hepatitis is not uncommon and from 1951 to 1954, 134 clinically diagnosed cases were seen at Baragwanath Hospital. Cases of acute massive necrosis were also seen in which the histology was consistent with fulminating viral hepatitis. In many livers in this region with primary cancer, the pathological picture of the associated cirrhosis was consistent with being the sequel to infective hepatitis. Further we have observed cases of infective hepatitis as demonstrated at biopsy in which liver cancer has later been found at post-mortem (Fig. 11). While this disease has not yet been proved to be a carcinogenic stimulus the hypothesis has considerable support.

Although the levels of these stimuli may vary in the environment, many are widespread among communities in which liver cancer appears to be uncommon. It is reasonable, therefore, to suggest that if such stimuli are in fact implicated their effect depends on alterations in liver metabolism which render the organ susceptible to the action of external factors, a possibility which has been discussed by Gillman and Gillman (1951). Evidence for abnormalities in hepatic metabolism are discussed in the following section.

### *Hepatic Dysfunction in the Indigenous African Negro*

Evidence in man and animals suggests that an already damaged liver is more susceptible to hepatotoxic agents than a healthy liver. The pathological and physiological abnormalities observed in the livers of the local Bantu and their significance have been discussed in detail elsewhere (Higginson, Grobbelaar and Walker, 1956).

It would appear that while the liver histology, "liver function tests" and serum proteins are normal in African babies, irreversible abnormalities arise during the first years of life as compared to European infants. Further, all necropsy and "liver function" series show a high incidence of hepatic disorder in apparently healthy adult Africans which appears irreversible (Wayburne, Bersohn and Sussman, 1953 ; Walker and Arvidsson, 1954). Although these changes may only reflect relatively slight liver damage, and accordingly cannot *per se* be regarded as premalignant, we feel there is evidence to suggest that such livers may be unduly susceptible to hepatotoxic agents causing cirrhosis of the post-necrotic or premalignant type (Higginson, Grobbelaar and Walker, 1956). It is also possible as has been suggested by Davies (1949) that the hormonal background of the African Negro may be abnormal due to either relative or absolute hyperoestrogenation. This fact may be of significance in view of the effects of hormones in experimental hepatomas. While it is impossible to prove that the above abnormalities do actually sensitise the liver to carcinogenic agents, they provide evidence that the metabolism of the liver in the African Negro by Western standards is abnormal.

### ROLE OF MALNUTRITION

The number, cell type, and induction time of many experimental animal tumours can be considerably modified by dietary manipulation (Opie, 1944). Liver tumours have also been produced in rats by a choline deficient diet alone (Salmon and Copeland, 1954). Many communications accordingly on liver disease in Africa have implicated malnutrition, especially deficiencies of protein and B complex, as a direct cause of cancer (Berman, 1951). On the other hand, there is little direct evidence to show that deficiencies of those food factors, the absence of which causes liver disease in animals, are involved in human liver disease (Higginson, Grobbelaar and Walker, 1956). In man, only in kwashiorkor, a nutritional disease of infancy, have the liver lesions been definitely proved to be related to malnutrition. There is no satisfactory support for the view that the post-necrotic cirrhosis of the adult South African Bantu is a result of this disease or any other specific dietary deficiency. Furthermore, in the adult Bantu Negro, cirrhosis following severe fatty change is very rare, a fact which would suggest that a deficiency of lipotropic agents is not important.

While the metabolic abnormalities in infancy, described above, may be the result of sub-clinical malnutrition, those seen in adult life appear irreversible and do not disappear in an improved dietary context (Wayburne, Bersohn and Sussman, 1952, 1953). The geographical distribution of malnutrition even in infancy, is not identical with that of liver carcinoma. Hepatic carcinoma appears rare in India (Khanolkar, 1951) although malnutrition is widespread (Thomson, 1946). In Jamaica, where nutritional deficiencies in both children and adults may be marked, liver cancer is uncommon (Bras, Brooks and Depass, 1955). Further, I have been unable to find reports of an increase of primary liver carcinoma from those parts of Europe where undernutrition was widespread in World War II. It is unlikely, therefore, that malnutrition whether of infancy or adult life, alone is capable of inducing liver tumour formation. If, as has been suggested, the metabolic disorders of infancy predispose the liver to carcinogens in later life the role of malnutrition would, at the most, be indirect.

### CONCLUSION

While the pathogenesis of primary liver cancer in South Africa would appear to have many similarities to hepatocellular tumours produced by " butter yellow " in rats, the possibility and significance of irreversible changes in liver metabolism arising in infancy warrants further study. If, as is possible, these abnormalities sensitise the liver to carcinogens in later life, then prevention of this cancer is possible. The significance of child welfare in such regions accordingly assumes basic importance, since the identification and elimination of the extrinsic carcinogenic stimuli may prove difficult or impossible, especially if viral in origin.

### SUMMARY

Certain pathological and cytological features of liver carcinoma on the African continent are reviewed.

It is suggested that the distinction between hepatocellular and cholangiocellular forms may be of fundamental significance. The high incidence of liver cancer in the Bantu is mainly due to an increase in the former.

The relationship of cirrhosis to primary liver cancer is briefly discussed and it is emphasised that in Africa it is a nodular hyperplastic cirrhosis probably of post-necrotic origin which most frequently becomes malignant, in contrast to the type of cirrhosis described in Europe and North America. It is considered that parenchymal cell hyperplasia associated with cirrhosis is essentially precancerous.

It is suggested that the liver in the African native is damaged in childhood, thus rendering it more susceptible to a carcinogenic stimulus. The significance of malnutrition would accordingly be only indirect by sensitising the adult liver. The only carcinogenic stimulus as yet implicated in human liver cancer is infective hepatitis, although many other possibilities should be considered, especially native medicines.

Prevention would appear to be possible either by eliminating abnormalities in hepatic metabolism arising in infancy or by avoidance of the carcinogenic stimuli causing post-necrotic cirrhosis in later life.

Our thanks are due to Mr. M. Ulrich for the microphotographs.

### REFERENCES

Bergeret, C. and Roulet, F.—(1947) *Acta. trop., Basel*, **4**, 210.
Berman, C.—(1951) ' Primary Carcinoma of the Liver '. London (Lewis).
Bras, G. Brooks, S. E. H. and Depass, E. E.—(1955) *Docum. med. geogr. Trop.*, **7**, 146.
Cameron, G. R.—(1952) ' Pathology of the Cell '. London (Oliver & Boyd).
Cowdry, E. V.—(1955) ' Cancer Cells.' London (Saunders).
Davies, J. N. P.—(1949) *Brit. med. J.*, **2**, 676.—(1955) *J. nat. Cancer Inst.*, **15**, 1637.—(1952) *W. Afr. med. J.*, **1**, 1.
Edmondson, H. A. and Steiner, P. E.—(1954) *Cancer.*, **7**, 684
Firminger, H. I.—(1955) *J. nat. Cancer. Inst.*, **15**, 1427.
Gillman, J.—(1940) *S. Afr. med. Sci.*, **5**, 46.
*Idem* and Gillman, T.—(1951) ' Perspectives in Human Malnutrition '. New York (Grune & Stratton).
Grusin, H.—(1955) *S. Afr. med. J.*, **29**, 117.
Haddow, A.—(1955) *Ann. Rev. Biochem.*, **24**, 689.
Hauschka, T. S. and Levan, A.—(1953) *Exp. Cell. Res.*, **4**, 457.
Higginson, J.—(1951) *Cancer*, **4**, 1224.—(1955) *Schweiz. Z. Path.*, **18**, 625.
*Idem*, Grobbelaar, B. and Walker, A. R. P.—(1956) *Amer. J. Path.*, in Press.
*Idem* and De Meillon, B.—(1955) *Arch. Path.*, **60**, 341.

Hou, P. C.—(1955) *Schweiz. Z. Path.*, **18**, 657.
Howatson, A. F. and Ham, A. W.—(1954) *Cancer Res.*, **15**, 62.
Hunt, M. A. and Schoental, R.—(1952) *Rep. Brit. Emp. Cancer Campgn.*, **30**, 256.
Khanolkar, V. R.—(1951) *Acta. Un. int. Cancr.*, **7**, spec. no. 51.
Marsden, A. T. H.—(1955) *Schweiz. Z. Path.*, **18**, 644.
Miller, J. A. and Miller, E. C.—(1953) *In*: Greenstein, J. P. and Haddow, A., *Eds.* ' Advances in Cancer Research ', **1**, 339. New York (Academic Press).
Milne, L. S.—(1909) *J. Path. Bact.*, **13**, 127.
Muir, R.—(1908) *Ibid.*, **12**, 287.
Ninard, B.—(1950) ' Tumeurs du Foie '. Paris (Francois).
Oettlé, A. G.—(1956) *J. nat. Cancer Inst.*, **17**, 249.
*Idem* and Higginson, J.—(1956) *Ibid.*, **17**, 281.
Opie, E. L.—(1944) *J. exp. Med.*, **80**, 219 and 231.
Payet, M., Camain, R., Pène, P. and Guerin, J.—(1953) *Sem. Hôp. Paris*, **64**, 3230.
Price, J. M., Harman, J. W., Miller, E. C. and Miller, J. A.—(1952) *Cancer Res.*, **12**, 192.
Roulet, F. C.—(1951) *Schweiz. Z. Path.*, **14**, 237.
Salmon, W. D. A. and Copeland, D. H.—(1954) *Ann. N.Y. Acad. Sci.*, **57**, 664.
Thomson, A. M.—(1946) *Proc. Nutr. Soc.*, **5**, 62.
Walker, A. R. P. and Arvidsson, U. B.—(1954) *J. clin. Invest.*, **33**, 1358.
Walsh, J. M. and Wolff, H. H.—(1952) *Lancet*, ii, 1007.
Wayburne, S., Bersohn, I. and Sussman, L.—(1952) *Rep. S. Afri. Inst. med. Res.*, p. 54.—(1953) *Ibid.*, p. 20.
Weiler, E.—(1952). *Z. Naturforsch.*, **76**, 324